# A rare case of *Candida parapsilosis* osteomyelitis: a literature review and proposed treatment algorithm

**DOI:** 10.1186/s13037-017-0146-9

**Published:** 2017-12-21

**Authors:** John Michael Yingling, Li Sun, Richard Yoon, Frank Liporace

**Affiliations:** Division of Orthopaedic Trauma & Adult Reconstruction, Department of Orthopaedic Surgery, Jersey City Medical Center – RWJBarnabas Health, Jersey City, NJ 07302 USA

**Keywords:** Acute and chronic deep fungal infection management, Exposed hardware, Fungus, Irrigation and debridement, Negative pressure wound therapy, Removal of hardware, Saucerization, Ankle fracture

## Abstract

**Background:**

*Candida parapsilosis* is a rare opportunistic pathogen that can be found in immunosuppressed patients. There are seldom-reported cases of fungal osteomyelitis surrounding orthopedic implants.

**Case presentation:**

This is a case of chronic *Candida parapsilosis* osteomyelitis in an immunocompromised patient with a prior open reduction and internal fixation for a closed bimalleolar ankle fracture that went on to neglected wound complications. The patient underwent series of treatments including removal of hardware, serial irrigation and debridements, negative pressure wound therapy, and intravenous antifungal therapy. Our case illustrates the possibility of this rare pathogen involved in orthopedic surgery particularly in immunocompromised hosts.

**Conclusion:**

Fungal and atypical pathogens should always be considered in such patients or if another diagnosis is not clear. Protracted time to culture specimens should be considered for at least four weeks in such situations. This article outlines a review of the literature and treatment algorithm to guide physicians when managing patients with this rare infection.

## Background

*Staphylococcus aureus* is the most common infecting organism found in adults with osteomyelitis [1]. *Salmonella* osteomyelitis has long been associated with SS or SC hemoglobinopathies [2]. Fungal osteomyelitis is seen increasingly in chronically ill patients receiving long-term intravenous therapy or parenteral nutrition and the immunocompromised [3]. The most common fungal osteomyelitis pathogens are Candida species. *C albicans* is the most common species isolated in culture [4, 5]. Healthy subjects can harbor fungal pathogens on their skin, in their GI tract, sputum, or other body fluids, and can present with musculoskeletal fungal infections, but most only become pathogenic in a host with a depressed immune system [4, 6]. Most resources will agree Human Immunodeficiency Virus (HIV) infection, cirrhosis, cancer, diabetes, prolonged corticosteroid use, alcoholism, and renal failure are among predisposing factors for fungal infections [6]. Surprisingly, in one study of 207 cases of candida osteomyelitis, a majority of the patients were not heavily immunosuppressed (neutropenic, transplant patients, underlying hematologic malignancy etc.) [4] Risk factors for invasive candidiasis include recent surgery, intravenous drug abuse (IVDA), broad spectrum antibiotic use, chemotherapy, parenteral nutrition, and central venous catheters [3]. Candida osteomyelitis develops from hematogenous spread of infection in approximately 70% of cases and can present up to 3 years after bloodstream infection [5]. Direct inoculation occurs in about 24%, and contiguous infection in less than 10% of cases. The diagnosis can often be difficult. Traditional laboratory inflammatory markers such as white blood cell count (WBC), erythrocyte sedimentation rate (ESR), and C-reactive protein (CRP) levels could only be slightly elevated or even normal. Clinically, the majority of patients will present with pain, tenderness, erythema, and decreased functionality of the extremity, but rarely with fever or draining wound. Candida osteomyelitis is diagnosed by culture via direct biopsy in over 70% of cases [4]. Interestingly, in patients with disseminated candidiasis, only 30% to 50% have a positive blood culture, which shows the difficulty in isolating the organism by blood culture. [7] Fungal infections are often complicated by concomitant bacterial infections, *Staphylococcus aureus* being the most common, but also included is *Proteus mirabolis* [4]. Fluconazole has been used to successfully treat candida osteomyelitis in spine patients and is considered to have a good safety and tolerability profile. Voriconazole may be used for fluconazole resistant infections and is said to have good bone penetration [3, 8, 9]. Adding to the difficulty of treating Candida infections, some species, namely *albicans* and *parapsilosis,* share the ability to form biofilms that may be resistant to standard -azole antifungals. Importantly, Echinocandin agents like Caspofungin or Micafungin have a unique mechanism of action that allows them to be used in combination with other antifungals and may be effective against biofilms namely involved with retained hardware [3, 10–12]. Traditionally surgical intervention has involved debridement of all necrotic tissue including bone and sinus tracts with specimens sent for culture and pathology, irrigation with copious amounts of sterile fluid, and placement of antifungal and/or antibiotic beads if necessary. Based on the stability of the bone or ability to stabilize with external methods, such as splinting, all internal hardware may be removed [3, 4, 13]. 90% of patients have complete or at least partial response to treatment with surgery and antifungal therapy [4]. This is a report of a patient’s presentation, course, and treatment that will contribute to the paucity of literature on *Candida parapsilosis* Osteomyelitis.

## Case presentation

An adult African American female was admitted to our institution for altered mental status and hypotension in October of 2015. Exposed hardware on her left ankle was noted from a previous open reduction and internal fixation 21 years prior.

The patient lived in a nursing home and her past medical history included end stage renal disease requiring dialysis three times per week, HIV on Highly Active Antiretroviral Therapy (HAART) diagnosed 10 years earlier, Hepatitis B and C, hypertension, coronary artery disease status post internal defibrillator placement, congestive heart failure, history of cerebral vascular accidents, anemia of chronic disease, seizure disorder, and dementia. The patient was noted to have exposed hardware of her left ankle on visits as far back as 9 years prior to the discussed admission to our institution.

**Fig. 1:**
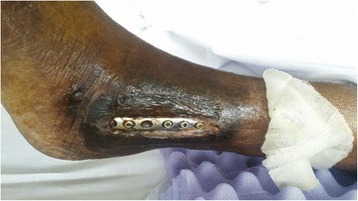
Left ankle displaying exposed hardware with no drainage or erythema

**Fig. 2:**
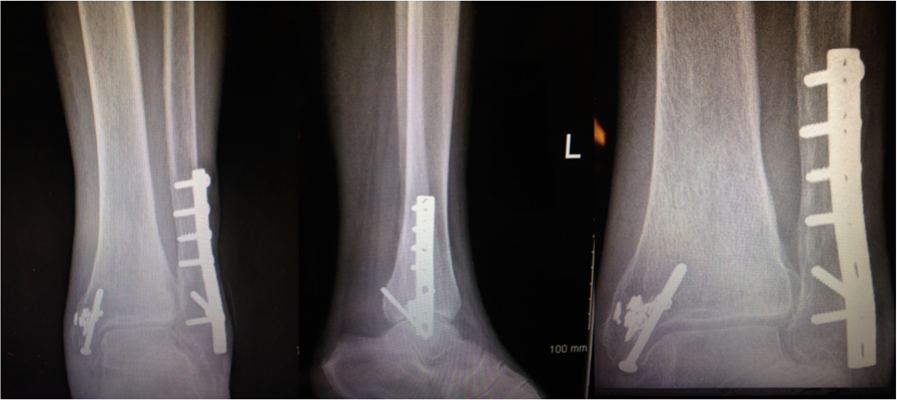
Preoperative AP, lateral, and mortise view of left ankle demonstrating retained hardware

**Fig. 3:**
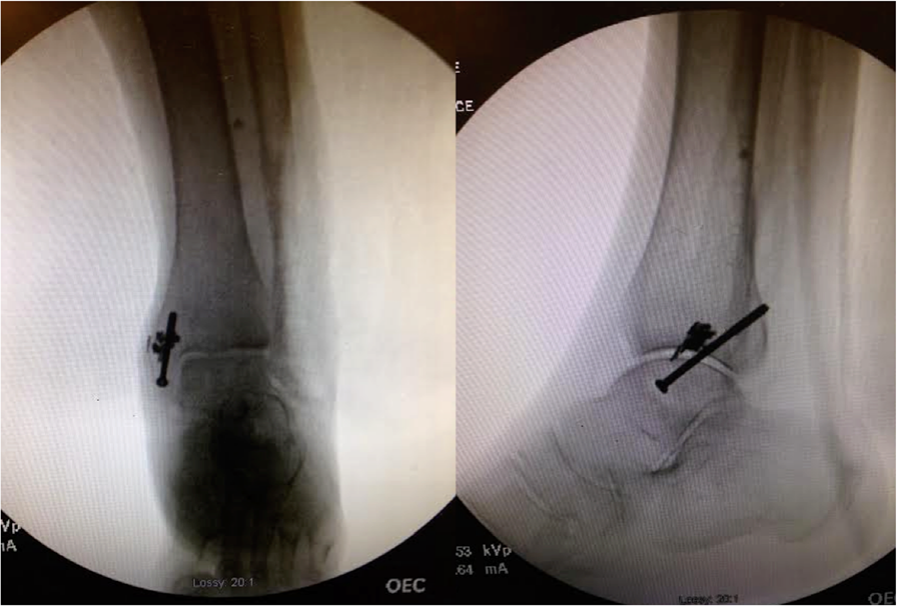
Intra-operative fluoroscopic images demonstrating the removal of fibular hardware and saucerization of the bone

During her initial work up the diagnosis of multilobular pneumonia was made and she was started on linezolid and aztreonam while initial blood cultures were negative. The patient went on to respiratory failure and septic shock secondary to her multilobular pneumonia, was subsequently intubated, and treated with vasopressors and antibiotics. The following day her sputum cultures grew *Candida albicans* and fluconazole was added to her treatment. Eight days later the patient showed improvement and was extubated still requiring vasopressors and hemodialysis. Clindamycin was added to the antibiotic regiment. Six days later Orthopedics was consulted to evaluate the exposed hardware of the lateral incision site of her left ankle (Fig. 1). The wound was dry, showed no signs of acute infection (erythema, purulent drainage, fluctuance, or warmth). The Radiographs showed retained medial and lateral hardware (Fig. 2). The following day the patient was taken to the operating room for removal of the exposed fibular plate and screws and a thorough irrigation and debridement of necrotic tissue. Intra-operative samples of peri-implant tissues and bone were sent for gram stain and culture. A negative pressure wound therapy dressing (NPWT) (V.A.C. Ulta Negative Pressure Wound Therapy System, KCI San Antonio, TX) was placed sterilely in the operating room. The NPWT dressing was changed at bedside every two days. The intraoperative bone sample cultures grew *Candida parapsilosis* and the soft tissue cultures grew *Candida parapsilosis* and *Proteus Mirabilis*. The NPWT dressing was then discontinued and the wound was dressed with sterile 4 × 4 gauze soaked with 0.25% Dakin’s solution. Under the direction of our infectious disease specialist the fluconazole was continued for coverage of both candida species. A week later the patient was taken to the operating room for another irrigation and radical debridement of necrotic bone, saucerization, and wound closure. Intra-operative fluoroscopic images are shown in (Fig. 3). A NPWT dressing was placed with scheduled changes on floor every three days. A variable course treating the patient’s sepsis and multiple medical issues complicated by bilateral upper extremity deep vein thrombosis ensued. The patient was intubated and transferred to the Intensive Care Unit on multiple vasopressors for closer monitoring after pulseless apneic episodes. At this time Caspofungin was added to her antifungal regiment of fluconazole along with other stronger broad-spectrum antibiotics. The patient eventually expired from her medical comorbidities.

## Discussion

**Fig. 4:**
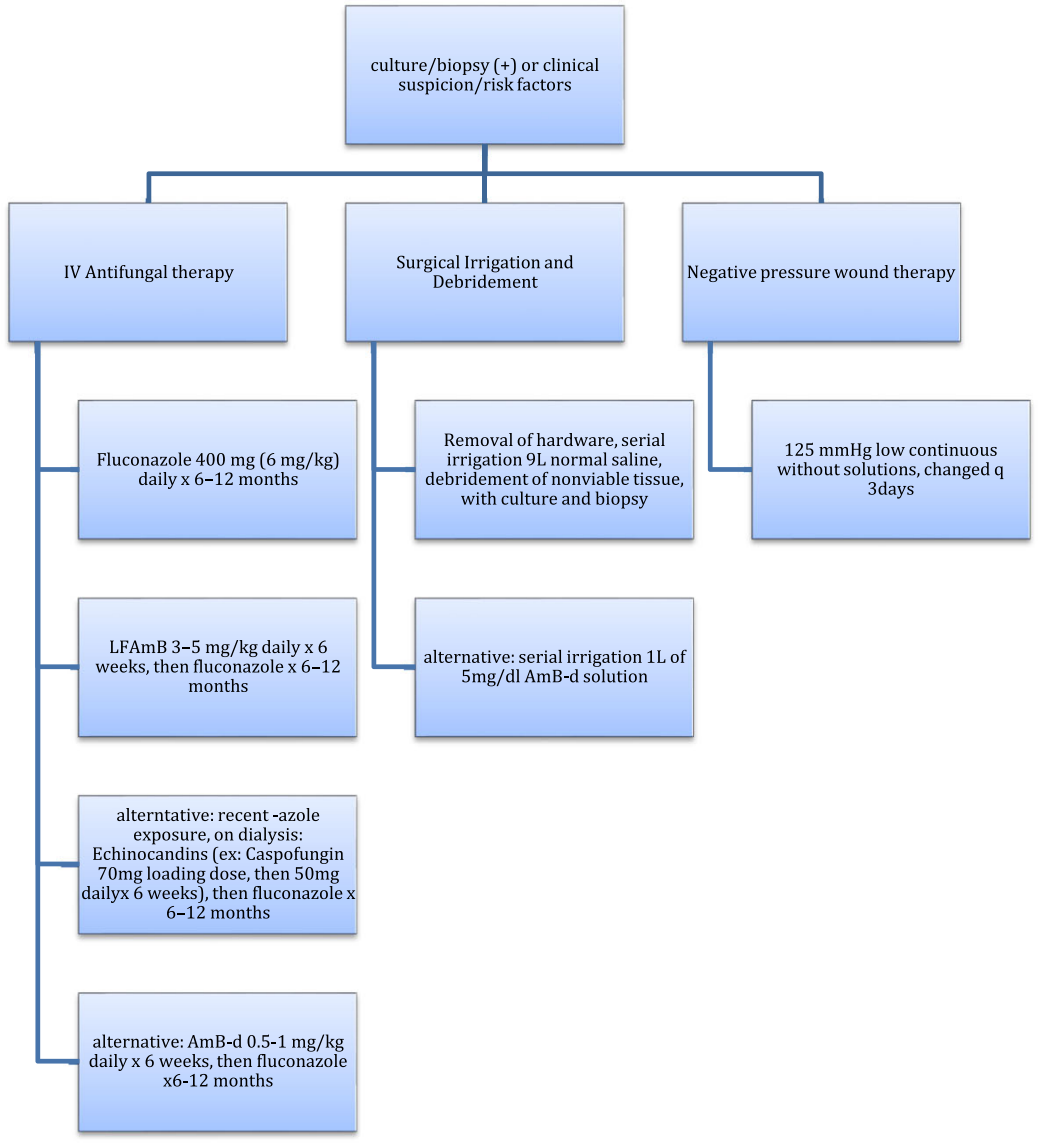
Algorithm for treating peri-implant *Candida parapsilosis* osteomyelitis

Candida osteomyelitis has been described in the literature primarily as *Candida albicans* species. This rare case of *Candida parapsilosis* osteomyelitis in an immunosuppressed patient is one of the few reported cases to be found. It is important to bring this to clinicians’ attention in order to keep it as differential diagnosis when treating similar patients in the future. Fungal osteomyelitis is difficult to diagnose in any patient with retained orthopedic hardware. Patients may often present with an equivocal clinical picture, normocytic labs, normal vital signs, unchanged radiographs, and wounds with minimal drainage. During the initial work up of a patient with negative blood cultures and risk factors for fungal infections you must maintain a high clinical suspicion for fungal osteomyelitis. It is important to keep fungal cultures for up to 4 weeks, as they may not initially demonstrate a pathogen [14]. The current recommendations of the Infectious Disease Society of America (IDSA) recommend 6-12 months of systemic antifungal agents in conjuncture with surgical debridement usually in the form of Fluconazole 400 mg (6 mg/kg) daily for 6–12 months. Lipid formulation of Amphotericin B 3–5 mg/kg daily for several weeks, then fluconazole for 6–12 months is also recommended, or an Echinocandin or Amphotericin B-d 0.5–1 mg/kg daily for several weeks then fluconazole for 6–12 months as an alternative, with the European recommendations being similar [12, 15–17]. Echinocandins have only parenteral routes of administration but have a very safe profile and do not need dosing for renal impairment or dialysis as they are eliminated through non-enzymatic degradation [17]. If financial or time constrains arise it may be reasonable to administer a shorter duration of IV antifungal therapy (AFT) as long as you ensure a thorough surgical debridement. Miller et al. described a mean duration of 45 days of IV AFT with surgical debridement in twenty-three cases and reported 90% good outcomes with no relapsing infections. Concomitant bacterial infection may convolute the picture but should be treated with culture sensitivity and specificity guided IV antibiotics along with the AFT. Due to the ability of Candida species to form biofilms it is important to remove any retained implants if the stability of the bone is adequate or maintained by other means such as splinting. Some have proposed using 1 L of 5 mg/dl Amphotericin B Deoxycholate irrigation solution with continuous suction without subsequent saline washes. They reported no complications or systemic toxicity but also have no comparisons in their study [16]. In the interim we used 0.25% Dakin solution, or Sodium Hypochlorite, as a topical antiseptic adjunct. Dakin solution, though controversially may have negative effects on neutrophil chemotaxis and fibroblast viability, is bactericidal and can be useful in the setting of an immunocompromised patient [18–20]. In order to enhance the penetration of IV AFT and blood flow to the infected area, a thorough surgical removal of all unviable bone and soft tissue from the area is recommended. This usually will require serial debridement at time intervals often dictated by the patient’s ability to tolerate surgery. During the initial and all subsequent procedures it is important to obtain culture samples along with tissue biopsy for histologic evaluation and monitoring for eradication of the pathogen as blood culture, laboratory markers, and clinical signs may be inconsistent and unreliable often due to the patient’s inability do respond. Based on current literature and the personal experience at our institution we propose the following algorithm for treating peri-implant *Candida parapsilosis* osteomyelitis in the Fig. 4 provided.

## Conclusion

Fungal and atypical pathogens should always be considered in such patients or if another diagnosis is not clear. Protracted time to culture specimens should be considered for at least four weeks in such situations. Treatment should consist of early antifungal medication, serial irrigation and debridement, and negative pressure wound therapy using the proposed algorithm.

## Abbreviations

AFT: Antifungal therapy
CRP: C-reactive protein
ESR: Erythrocyte sedimentation rate
HAART: Highly Active Antiretroviral Therapy
HIV: Human Immunodeficiency Virus
IDSA: Infectious Disease Society of America
IVDA: Intravenous drug abuse
NPWT: Negative pressure wound therapy dressing
WBC: White blood cell count
